# Stromal Cell Subsets Directing Neonatal Spleen Regeneration

**DOI:** 10.1038/srep40401

**Published:** 2017-01-09

**Authors:** Jonathan K. H. Tan, Takeshi Watanabe

**Affiliations:** 1 AK Project, Graduate School of Medicine, Kyoto University, Kyoto 606-8501, Japan; 2 Division of Biomedical Science, Research School of Biology, The Australian National University, Canberra 0200, Australia

## Abstract

Development of lymphoid tissue is determined by interactions between stromal lymphoid tissue organiser (LTo) and hematopoietic lymphoid tissue inducer (LTi) cells. A failure for LTo to receive appropriate activating signals during embryogenesis through lymphotoxin engagement leads to a complete cessation of lymph node (LN) and Peyer’s patch development, identifying LTo as a key stromal population for lymphoid tissue organogenesis. However, little is known about the equivalent stromal cells that induce spleen development. Here, by dissociating neonatal murine spleen stromal tissue for re-aggregation and transplant into adult mouse recipients, we have identified a MAdCAM-1^+^CD31^+^CD201^+^ spleen stromal organizer cell-type critical for new tissue formation. This finding provides an insight into the regulation of post-natal spleen tissue organogenesis, and could be exploited in the development of spleen regenerative therapies.

Spleen is an organ intimately associated with blood filtration. It broadly acts in a two-fold manner to remove damaged or senescent red-blood cells, and to detect and respond to blood-borne pathogens^1^. As an immune organ, the capacity for spleen to filter blood means that pathogens or antigens entering the marginal zone (MZ) are effectively screened, enabling immediate innate or longer-lasting adaptive immune responses. This is facilitated by numerous immune cell types including macrophages^2^, monocytes^3^, dendritic cells (DC)^4^ and T and B cells located in the MZ, red pulp (RP) and white pulp (WP).

Establishment of organized spleen structure is essential for effective immune responses^5^. White pulp compartmentalization is organized by stromal cells, which direct hematopoietic cell populations into distinct areas of spleen. In white pulp, well-defined stromal cell populations include follicular dendritic cells (FDC) and fibroblastic reticular cells (FRC), which organize B cell and T cell compartments, respectively^6^. The marginal zone which encircles white pulp contains a stromal layer of marginal zone reticular cells (MRC)^7^, that is most prominent adjacent to B cell follicles, but interrupted at MZ bridging channels where the marginal sinus connects directly to T cell areas^6^. Stromal tissues are not passive bystanders, with evidence that lymph node FRC populations contribute directly to the attenuation of T cell responses^8^. Furthermore, spleen stromal tissues can direct the development of stem cell progenitors towards antigen-presenting cell lineages^9^,^10^, and change the behavior of inflammatory DC into a regulatory phenotype^11^.

Stromal cells are also essential for lymphoid tissue organogenesis. Termed lymphoid tissue organizers, these stromal cells interact through lymphotoxin-β receptor (LTβR) engagement with lymphotoxin-α_1_β_2_ (LTα_1_β_2_) expressed on lymphoid tissue inducer (LTi) cells, to initiate embryonic LN development^12^. The cascade of events leading from anlagen to lymphoid tissue formation have been well-described^13^, where maturation of local mesenchymal stromal cells into LTo via LTα_1_β_2_ signaling^14^ leads to expression of adhesion molecules and chemokines critical for hematopoietic cell recruitment and tissue development. Moreover, LTo not only function in LN embryogenesis, but their activities have also been implicated in tertiary^15^ or artificial lymphoid tissue formation^16^,^17^. The equivalent stromal cells directing spleen development, however, remain unknown. Identification of such spleen organizer cells would be essential in designing specific strategies for spleen tissue regeneration. Here, we describe a murine model for spleen cell-aggregate graft transplantation, and report the isolation of a defined spleen stromal population that is essential for regeneration of neonatal spleen tissue.

## Results

### Establishment of a Spleen Cell Aggregate Transplant System in Mice

**Figure 1:**
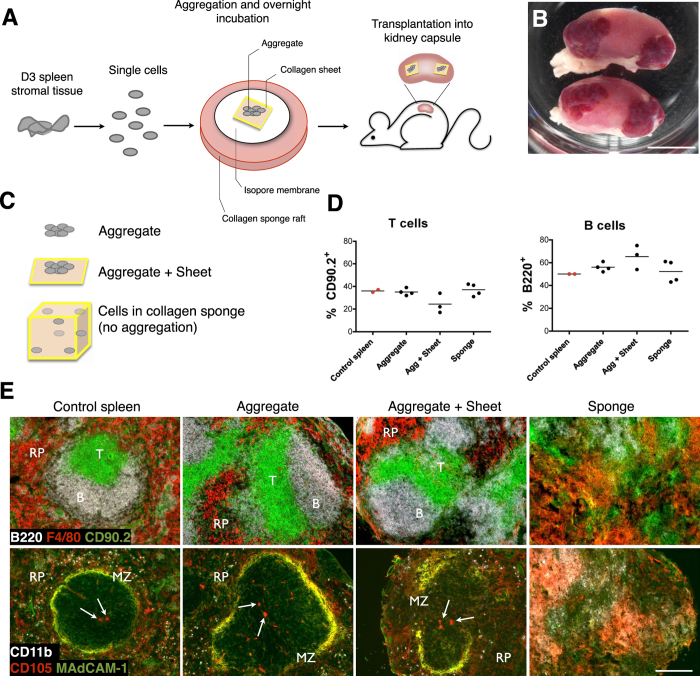
Development of spleen tissue from 3 day-old spleen stromal cell aggregates. (**A**) Schematic diagram of aggregate transplantation. Spleen stromal tissues from day 3 (D3) postnatal mice were isolated and enzymatically digested into a single-cell suspension. Cells were then aggregated and aliquoted on top of a collagen sheet resting over an isopore membrane/collagen sponge complex immersed in cell culture medium. Following overnight incubation, cell aggregate/collagen sheet constructs were transplanted under the kidney capsule of adult splenectomised recipient mice. (**B**) Macroscopic appearance of regenerated spleen tissue 4 weeks post-transplantation. Scale bar, 5 mm. (**C**) To optimize graft construction, cells derived from D3 spleen stromal tissue were aggregated and transplanted alone or above collagen sheets, or collagen sponges were loaded with equivalent cell numbers but omitting cell aggregation. Transplantation of all graft constructs were performed independently a minimum of 2 times, with 2–8 grafts/experiment. (**D**) Percent T and B cells in each construct 4 weeks post-transplantation was assessed by flow cytometry. Age-matched non-surgery mouse spleen was used as a control. (**E**) Spleen structure of 4 week grafts or control spleen visualized by immunofluorescence microscopy (RP, red pulp; MZ, marginal zone. Arrows indicate central arterioles). Original magnification 100×. Scale bar, 200 μm.

Lymphoid cell aggregation and transplantation has previously been demonstrated to represent a viable model for LN development^12^. To adapt this protocol and investigate spleen regeneration, we isolated spleen from neonatal 3 day-old (D3) mice and enzymatically digested splenic stromal tissues into a single-cell solution. Cells were then re-aggregated, loaded over a collagen sheet, and transplanted into the kidney capsule of adult splenectomized recipient mice (Fig. 1A). Consistent with bulk spleen stromal tissue preparations^18^, grafts constructed from aggregated neonatal spleen cells retained the capacity to develop gross spleen tissue (Fig. 1B). Since artificial lymph nodes (aLN) have been previously synthesized using stromal cells loaded onto a collagen sponge^16^, we assessed whether D3 neonatal spleen cell-loaded sponges would sustain tissue formation, alongside control aggregate-sheet constructs and non-scaffold supported aggregates (Fig. 1C). All grafts after 4 weeks displayed an influx of lymphocytes with percent T cells and B cells similar to native control spleen (Fig. 1D). However, only cell-aggregated grafts with or without collagen sheet support showed evidence of normal spleen structure, with collagen sponge grafts failing to organize spleen tissue (Fig. 1E). Cell to cell contact and paracrine signaling facilitated by cell aggregation therefore appears critical for spleen regeneration. In contrast, inflammatory stimuli required for aLN synthesis through enforced stromal lymphotoxin expression and addition of activated DC^16^ appear unnecessary factors for spleen regeneration, a reflection of secondary rather than tertiary lymphoid organogenesis. In all further experiments, aggregate-sheet constructs were used in favor of aggregates alone due to a greater level of mechanical support and graft stability during transplantation.

### Stromal Cells Contribute Exclusively to Complete Spleen Tissue Formation

**Figure 2:**
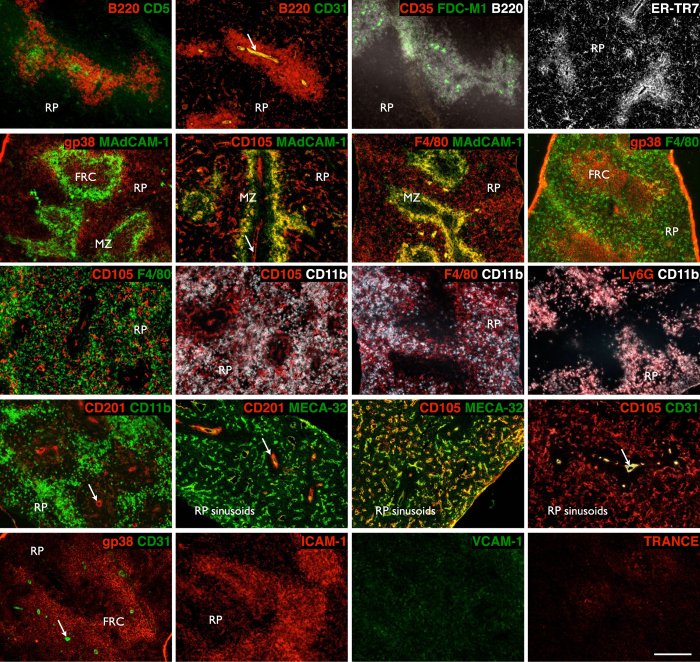
Stromal and lymphoid composition of early neonatal spleen. 3-day old spleen was cryosectioned and stained with indicated markers before immunofluorescence analysis to differentiate tissue micro-architecture (RP, red pulp; MZ, marginal zone; FRC, fibroblastic reticular cells. Arrows indicate central arterioles). Original magnification 100×. Scale bar, 200 μm.

**Figure 3:**
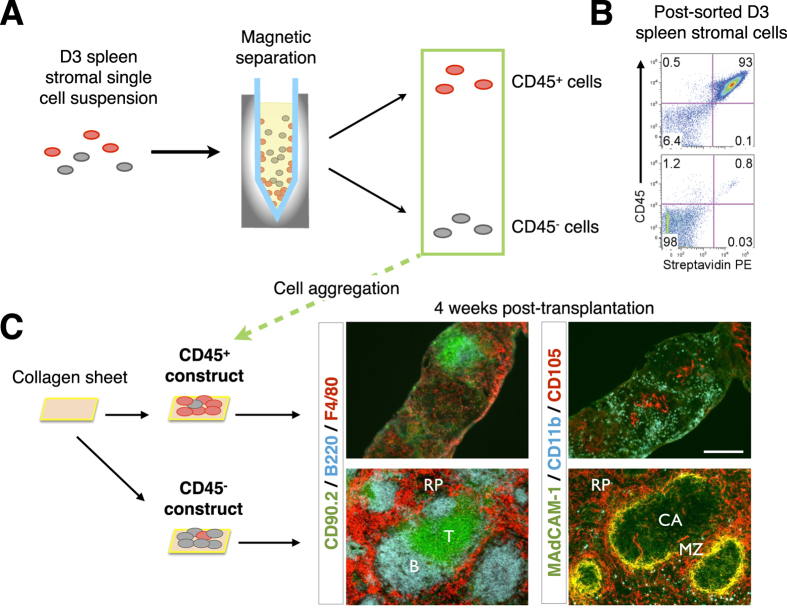
Spleen tissue regeneration from neonatal spleen stromal cell aggregates occurs independently of donor-derived hematopoietic cells. (**A**) D3 spleen stromal tissue was digested into a single-cell suspension and magnetically separated using biotin-conjugated anti-CD45 antibodies and anti-biotin microbeads into marker positive and negative fractions. (**B**) Separation purity was assessed by a flow cytometer using APC-eFluor780-conjugated anti-CD45 antibody and streptavidin-PE secondary reagent. Numbers in quadrants indicate percent cells. (**C**) CD45^+^ and CD45^−^ cell fractions were aggregated and placed over collagen sheets before transplantation and 4 week analysis by immunofluorescence microscopy using indicated markers (original magnification 100×, Scale bar, 200 μm). RP, red pulp; MZ, marginal zone; CA, central arterioles. Data are representative of two independent experiments.

A protocol for graft construction using single-cell digested, and re-aggregated spleen stromal tissue allows manipulation of individual cell populations within grafts, which in turn permits delineation of organizer cells responsible for spleen neogenesis. To first identify candidate organizer cells, we examined the cell composition of neonatal D3 spleen tissue by immunofluorescence staining (Fig. 2) and flow cytometry analysis (Supplementary Table S1). The contribution of distinct populations towards spleen neogenesis was then screened by a series of single-marker magnetic cell separations, followed by graft aggregation and transplant. To confirm that stromal but not contaminating hematopoietic cells in spleen tissue preparations controlled *de novo* spleen formation, single-cell suspensions labeled with biotin-conjugated anti-CD45 antibody were magnetically separated into CD45^+^ and CD45^−^ spleen fractions (Fig. 3A), with separation purity confirmed by flow cytometry (93% CD45^+^ cells and 98% CD45^−^ cells, respectively; Fig. 3B). Each fraction was then aggregated and placed over collagen sheets before transplant. A semi-quantitative assessment of spleen structure including formation of RP, multiple WP follicles displaying T and B cell segregation, and MZ, revealed that complete spleen regeneration could only be achieved using CD45^−^ stromal cell aggregates (Fig. 3C and Table 1). While tissue was also recovered from CD45-enriched aggregate grafts, these clearly lacked definitive spleen structure.

### Selective Enrichment of Individual Stromal Cell Populations Identifies Markers Associated with Graft Formation

**Figure 4:**
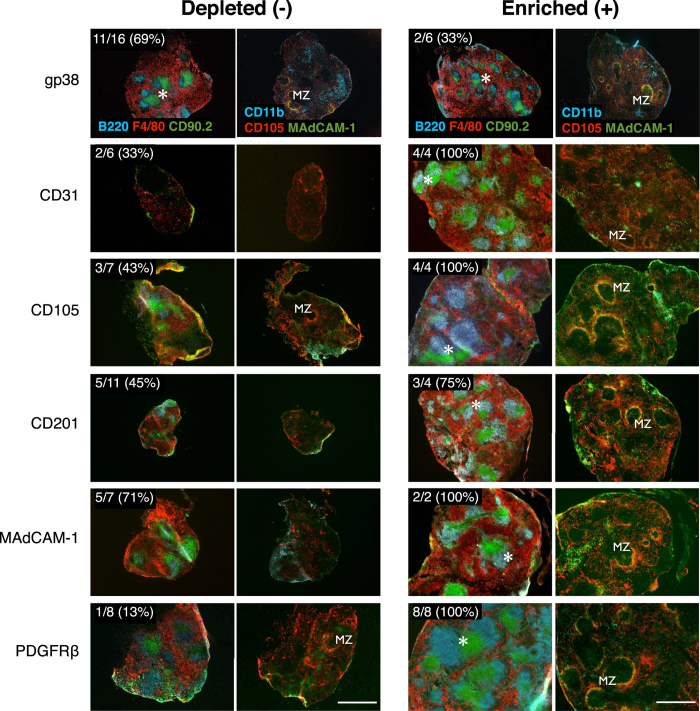
Spleen tissue regeneration of aggregate grafts prepared by magnetic depletion or enrichment of stromal cell subsets. Aggregates were depleted (−) or enriched (+) of cells based on single markers before transplantation and assessment of tissue regeneration after 4 weeks by immunofluorescence microscopy. Representative images of graft development from cell marker depleted or enriched aggregates showing comparative efficiencies in formation of full spleen tissue architecture. Top left inserts show enumeration of total grafts recovered. Asterisks indicate organized T/B cell white pulp follicles; MZ, marginal zone. Original magnification 40×. Scale bar, 500 μm.

To screen for stromal cell populations carrying organizer activity, single-marker magnetic cell separation and aggregate transplants were performed using a panel of stromal markers (Table 1). Magnetic-based separations were limited by low target cell enrichment and the inability to exclude contamination from other cell populations (Table 1). However, as a screen comparing the relative capacity of single-marker enriched or depleted aggregates to regenerate tissue, a >10 fold-change between negative and positive target cells was consistently achieved. The sensitivity of this assay to report even small sub-population target cell changes was demonstrated by CD31, CD105, CD201, MAdCAM-1 or PDGFRβ enriched, but not depleted aggregates, robustly developing spleen tissue with distinct regions of red pulp, white pulp (displaying multiple follicles/segregated T and B cell areas), and marginal zones (Fig. 4). Consistent with trace tissue recovery from CD45-depleted aggregates (Fig. 3C), variable recovery of CD31, CD105, CD201, MAdCAM-1 or PDGFRβ depleted aggregates was also observed (Fig. 4), and these grafts corresponded with poor spleen compartmentalization. Overall, this screen generated a stromal marker profile which correlated with robust spleen regenerative capacity.

### CD31^+^CD105^+^CD201^+^MAdCAM-1^+^ Stromal Cells in Spleen and are Specifically Required to Induce Tissue Regeneration

**Figure 5:**
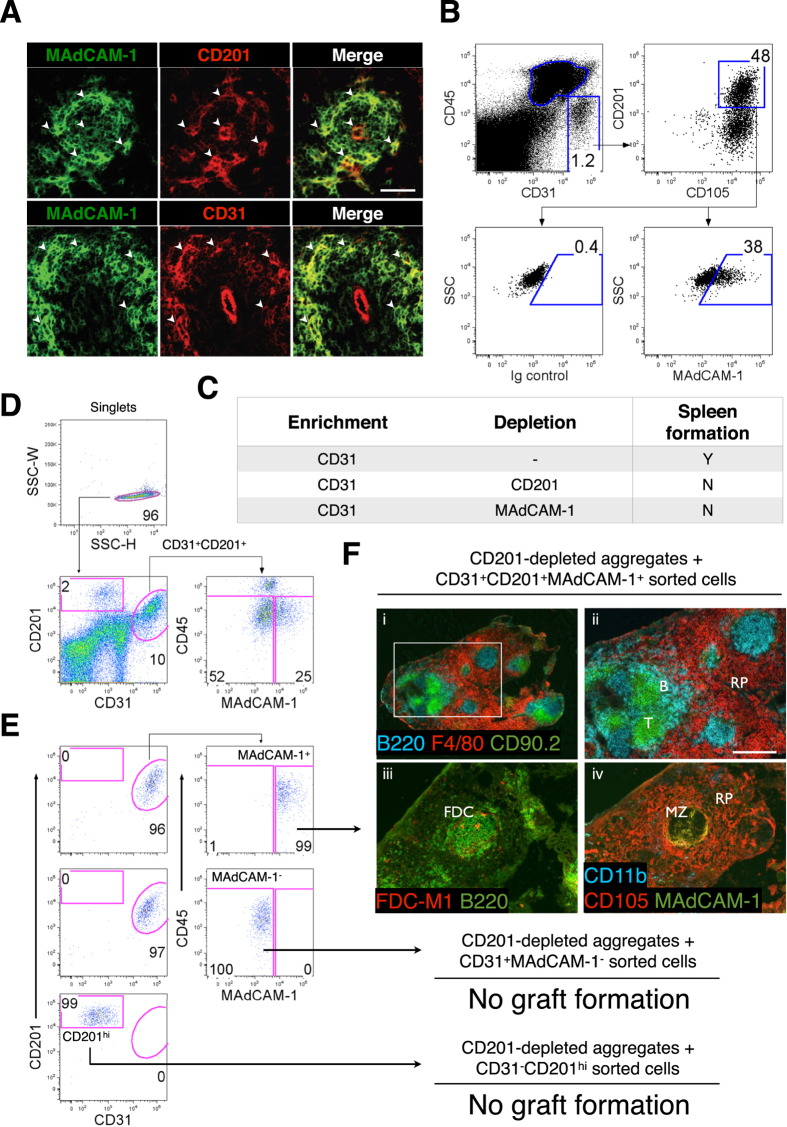
Neonatal CD45^−^CD31^+^CD105^+^CD201^+^MAdCAM-1^+^ cells represent spleen organizers. Cell markers shown to enrich for spleen forming activity (Table 1) were used to (**A**) assess D3 neonatal spleen sections for stromal cells co-expressing MAdCAM-1 and CD201 or CD31 (arrowheads; scale bar, 50 μm), and (**B**) identify a single CD45^−^CD31^+^CD105^+^CD201^+^MAdCAM-1^+^ stromal population by flow cytometry. (**C**) CD31^+^ aggregates from D3 spleen capable of tissue regeneration were depleted of CD201 or MAdCAM-1 expressing cells before aggregation and transplantation to confirm whether spleen forming activity segregated with CD31^+^CD201^+^ and CD31^+^MAdCAM-1^+^ populations. Formation of spleen tissue was assessed 4 weeks post-transplant. Each separation and transplantation was performed twice (2–3 grafts/experiment). (**D**) To ascertain *bona fide* spleen organizers, CD45^−^CD31^+^CD201^+^MAdCAM-1^+^ cells gated to exclude doublets were sorted by FACS and the purity verified (**E**). CD45^−^CD31^+^CD201^+^MAdCAM-1^−^ and CD45^−^CD31^−^CD201^hi^ cells were sorted in parallel as negative controls. (**F**) Sorted populations were re-added to aggregates depleted of CD201^+^ spleen-forming cells before transplantation. Tissues collected after 4 weeks were analyzed by immunofluorescence staining with indicated markers (ii, iii and iv, 100×). Scale bar, 200 μm. Enlargement of (i) (40×) is shown in (ii). RP, red pulp; MZ, marginal zone; FDC, follicular dendritic cells. No graft formation indicates the absence of trace spleen tissue. Data are representative of two independent experiments.

Based on the above marker profile, a single stromal cell population bearing comparable markers was sought *in vivo*. Stromal cells co-expressing MAdCAM-1 and CD201 or CD31 were evident in the marginal zone of D3 spleen (Fig. 5A), and further flow cytometric analysis confirmed a specific CD45^−^CD31^+^CD105^+^CD201^+^MAdCAM-1^+^ stromal population representing putative spleen organizer (SPo) cells (Fig. 5B). To determine whether tissue organizer potential segregated with SPo, two-marker magnetic cell separation and aggregation was performed. CD31-enriched aggregates normally competent for spleen regeneration (Table 1) were depleted of either CD201^+^ or MAdCAM-1^+^ cells, leading to abrogation of spleen development (Fig. 5C). Collectively, these results supported the segregation of spleen organizer activity with CD31^+^CD201^+^MAdCAM-1^+^ cells.

To conclusively demonstrate that this candidate population represented spleen organizers, CD45^−^CD31^+^CD201^+^MAdCAM-1^+^ cell singlets were sorted by FACS to high purity (>95%; Fig. 5D,E), before re-addition to CD201-depleted filler aggregates lacking spleen-forming potential. As controls, CD45^−^CD31^−^CD201^hi^ (CD201^hi^) and CD45^−^CD31^+^CD201^+^MAdCAM-1^−^ (MAdCAM-1^−^) cells were sorted in parallel. Replacement of both MAdCAM-1^−^ and CD201^hi^ cells to filler aggregates failed to rescue spleen development (Fig. 5F). However, addition of SPo cells restored full spleen regeneration capacity, demonstrating the specificity by which these cells function, and confirming that SPo represent *bona fide* spleen tissue organizer cells that are essential for neonatal spleen organogenesis.

### SPo Express Genes Associated with Tissue Organization But Not Mesenchymal Cells

**Figure 6:**
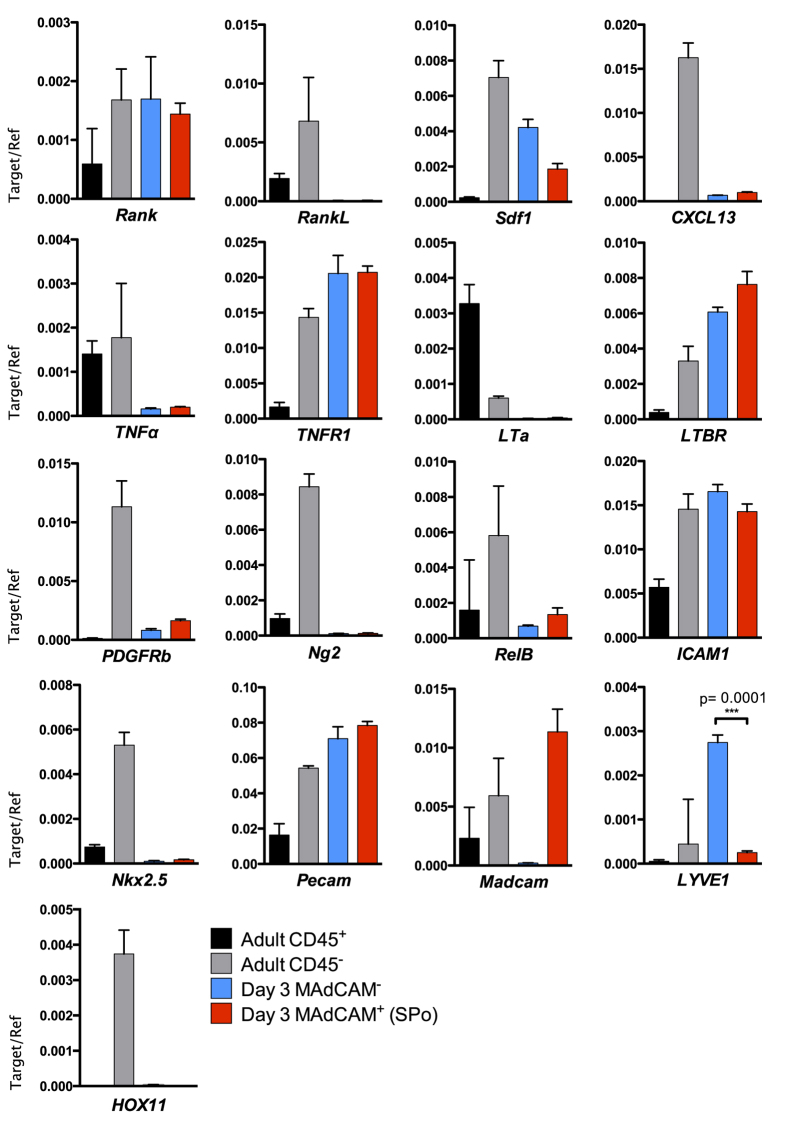
Relative abundance of mRNA transcripts in spleen organizer (SPo) cells. To determine gene expression between CD45^−^CD31^+^CD201^+^MAdCAM-1^+^ (MAdCAM-1^+^) SPo and CD45^−^CD31^+^CD201^+^MAdCAM-1^−^ (MAdCAM-1^−^) cells that do not induce spleen organogenesis, each population was sorted to purity from D3 spleen and assessed for the expression of mRNA by real-time quantitative PCR. CD45^+^ hematopoietic or CD45^−^ stromal cells enriched from adult spleen were used as controls. Gene expression was normalized to *β-actin. n* = 3, error bars indicate SEM. ***P<0.001; unpaired two-tailed *t* test.

Spleen organizer cells express MAdCAM-1 which is consistent with classically-described LTo, yet co-expression of CD31, CD105 and CD201 indicates cells of a mixed endothelial/mesenchymal lineage. Indeed, LN vascular endothelial cells expressing MAdCAM-1^19^, and CD105 or CD201 expression by mesenchymal stem or progenitor cells^20^,^21^,^22^ and vascular endothelial cells (Fig. 2), further exemplify the uncertainty surrounding cell lineage determination. To first confirm the validity of our sorting protocol, quantitative real-time PCR was used to demonstrate specific *Madcam* expression in SPo, but not CD45^−^CD31^+^CD105^+^CD201^+^MAdCAM-1^−^ D3 spleen stromal cells (Fig. 6). As expected, *Pecam* (CD31) was detected in both cell populations. However, expression of *Nkx2.5* or *HOX11* defining spleen mesenchymal lineages^23^,^24^ was not observed. The absence of these transcription factors suggests SPo are distinct from mesenchymal Nkx2.5^+^ cells with organizer activity found in embryonic spleen^23^.

Supporting a functional role as tissue organizers, neonatal SPo expressed high levels of *LTBR* but not *LTa*. This is consistent with previous findings that neonatal spleen stromal grafts require lymphotoxin signaling to functionally regenerate^18^. *TNFR1*, necessary for adult spleen lymphoid compartment formation^25^, was also expressed by SPo. While we detected *Rank* (TRANCE-R) expression as previously described on LN stroma^19^, *RankL* (TRANCE) expression characteristic of LTo was absent. This result was however consistent with a general lack of surface staining on D3 spleen sections (Fig. 2). Similarly, *CXCL13* normally produced by LTo was absent in SPo, in line with weak CXCL13 expression observed during neonatal spleen development^7^. Low level *Sdf1* (CXCL12) expression was however evident. Interestingly, differential *LYVE1* expression was observed between MAdCAM-1^+^ SPo and phenotypically similar but functionally non-organizing CD31^+^MAdCAM-1^−^ stromal cells, serving as a molecular marker to distinguish these cell subsets. LYVE-1 is normally associated with lymphatic endothelium^26^ or splenic platelets and megakaryocytes^27^. While lymphatic endothelium is not observed normally in spleen, transcriptional level expression may indicate that CD31^+^MAdCAM-1^−^ cells are precursor to LYVE-1^+^ vascular structures observed in the spleen of *Nkx2.3*^−/−^ mice^27^.

Mesenchymal lineage pericytes have been implicated in spleen tissue remodeling following lymphocytic choriomeningitis infection^23^, however a relationship between SPo and pericytes could not be established given a lack of both *Ng2* and *PDGFRb* expression (Fig. 6). Interestingly, under grafting and tissue regeneration conditions, PDGFRβ-enriched aggregates yielded a higher capacity for tissue formation compared with grafts depleted of PDGFRβ^+^ cells (Fig. 4). To determine whether PDGFRβ^+^ cells overlap in identity with SPo, flow cytometry analysis was performed but revealed no surface CD31 expression (Supplementary Fig. S1A), confirming PDGFRβ^+^ cells represent a distinct stromal subset. Cells expressing PDGFRβ did, however, co-express CD201 at low levels. Thus, in CD201-depleted grafts rescued by addition of SPo (Fig. 5), it remains a formal possibility that CD201^lo^PDGFRβ^+^ cells avoided magnetic separation to interact with CD31^+^ SPo during graft formation. Indeed, re-evaluation of CD201-depleted stromal cells demonstrated a higher percentage of residual PDGFRβ^+^ cells compared with PDGFRβ-depleted stromal preparations (Supplementary Fig. S1B). When considered jointly, these findings indicate that both PDGFRβ^+^ and CD31^+^ SPo cell types may cooperate during neonatal spleen tissue regeneration.

## Discussion

Organizer cells in spleen have eluded prior definitive identification although marginal zone reticular cells have previously been considered potential SPo candidates. MRC were first described in adult spleen, analogous to marginal sinus-lining cells surrounding the white pulp^28^. In spleen, MRC are thought to represent adult-stage LTo since after birth, MAdCAM-1^+^ cells can be observed concentrated around the central arteriole before expanding to form characteristic ring-like structures^7^. In terms of a prospective role as neonatal MRC, MAdCAM-1^+^ cells isolated from D3 spleen are indeed required for the induction of spleen tissue formation (Table 1). Interestingly, aggregate grafts also require cells expressing CD31, reflective of an endothelial lineage. Indeed, MAdCAM-1^+^VE-cadherin^+^ or CD31^+^ vessels were found to be encircled by CD4^+^ LTi during embryonic development^29^,^30^ strengthening the notion that stromal cells bordering the mesenchymal and endothelial cell lineages may represent spleen organizers.

Identification of a lineage-mixed CD31^+^CD105^+^CD201^+^MAdCAM-1^+^ organizer cell population here does, however, put SPo at odds with LTo commonly thought to derive from a purely mesenchymal origin^14^,^31^. Not only do MAdCAM-1^+^ LTo in lymph nodes express PDGFRβ and gp38, recently identified LTo in adipose tissue, perivascular FDC precursors, and adult MRC all share similar expression patterns^31^,^32^,^33^. In contrast, neonatal SPo express CD31, CD105 and CD201, lacking PDGFRβ and gp38 expression. Furthermore, compared with embryonic spleen Nkx2.5^+^ organizers^23^, SPo responsible for spleen regeneration in the present study do not express *Nkx2.5*.

To reconcile these inconsistencies, we propose that neonatal SPo represent an endothelial, or a transitional endothelial-mesenchymal population arising from early mesenchymoangioblasts, previously reported to differentiate into both mesenchymal stem cell and endothelial lineages^34^. This branch in development may result in distinct mesenchymal and endothelial-like organizer cell lineages, capable of independently directing embryonic^23^ and neonatal spleen organogenesis, respectively. The requirement for a second population of PDGFRβ^+^ stromal organizer cells to initiate spleen regeneration is, however, consistent with mesenchymal lineage Nkx2.5^+^ organizers described by Castagnaro *et al*. Although we show evidence that PDGFRβ^+^ cells are important for spleen tissue neogenesis, the PDGFRβ^+^ population as a whole remains heterogeneous and is known to contain pericytes, vascular smooth muscle cells and FDC precursors^32^. As only a fraction of PDGFRβ^+^ cells are likely to represent true organizer cells, further studies are necessary to investigate their precise identity and contribution towards spleen organogenesis.

In summary, we have developed a spleen transplant model that facilitates the manipulation of graft cell input, and dissected spleen stroma to define a specific MAdCAM-1^+^CD31^+^CD201^+^ organizer cell population essential for neonatal spleen regeneration. The ability to shape the cell composition of grafts now enables the role of multiple stromal cell populations which guide spleen development to be investigated. If the minimal cell requirements for spleen regeneration can be identified, this may represent a starting point for regenerative therapies targeted at repairing or replacing spleen tissue.

## Experimental Procedures

### Mice

BALB/cCrSlc and Balb/cAnNCrl/Anu mice were purchased from Japan SLC, Inc. and Australian Phenomics Facility, Australian National University (ANU), respectively. Mice were housed under specific pathogen-free conditions and all methods carried out according to experimental protocols approved by the Kyoto University Medical School Animal Experiment Ethics Committee and ANU Animal Experimentation Ethics Committee.

### Spleen stromal cell aggregates

Spleens were asceptically removed from 3 day-old BALB/c mice and dissociated between two sterile microscope slides into PBS. The solution containing a mixture of suspendable hematopoietic cells and non-suspendable stromal tissue was filtered through a 100 μm cell strainer (Becton Dickinson), discarding the flow-through. Non-suspendable tissue was transferred into 2 ml supplemented DMEM (sDMEM)^35^ containing 1 mg/ml Collagenase IV (Invitrogen), 40 μg/ml DNaseI (Sigma) and 2% FBS (Gibco) for 20 min at 37 °C with constant stirring to obtain a single-cell suspension^18^,^26^. Incubation was repeated twice more with addition of medium containing 1 mg/ml Collagenase D (Roche), 40 ug/ml DNaseI (Sigma) and 2% FBS before filtration through a 100 μm cell strainer. Aggregates were formed by resuspending cells at 5 × 10^7 ^cells/ml and drawing 20 μl of cell solution into a pipette tip subsequently sealed with Parafilm^36^. Cells were pelleted before removal of Parafilm and aspiration of aggregated cells onto a collagen sheet layered over an Isopore membrane filter (Millipore) and collagen sponge raft immersed in 1 ml sDMEM for overnight culture at 37 °C (Fig. 1A). Aggregate/sheet constructs were transplanted under the kidney capsule of 7 week-old splenectomised BALB/c mice at the upper and lower poles of each kidney. After 4 weeks, transplants were collected and analyzed by FACS or immunofluorescence staining.

### Antibodies and secondary reagents

Antibodies used for cell separation, flow cytometry and immunofluorescence staining are listed in Supplementary Table S2. Secondary reagents are listed in Supplementary Table S3.

### Magnetic cell separation

Single-cell suspensions were labeled with appropriate biotin-conjugated antibodies and purified anti-CD16/CD32 Fc Block diluted in labeling buffer (2% FBS/2 mM EDTA in PBS) and incubated for 10 mins at 4 °C. Cells were washed once with 10-fold excess volume buffer and incubated with anti-biotin microbeads (Miltenyi Biotec). After a second wash, cells were resuspended in buffer and placed inside an EasySep Magnet (STEMCELL Technologies). After 10 mins, the magnet was inverted and supernatant containing unlabeled cells decanted into a fresh tube.

### Flow cytometry and cell sorting

For all flow cytometry and cell sorting, propidium iodide (PI; 100 μg/ml) was added to discriminate dead cells. Flow cytometry was performed with FACS CantoII or FACSVerse (BD Biosciences) and data analyzed with FlowJo software (TreeStar). Fluorescence-activated cell sorting was performed with FACSAria (BD Biosciences).

### Immunostaining

Lymphoid organs and transplants were embedded in Tissue-Tek O.C.T compound (Sakura Finetechnical Co., Ltd.) and frozen in liquid nitrogen. Seven micrometre-thick cryostat sections were prepared and placed on glass slides (Matsunami Glass Ind. Ltd.). Sections were stored at −80 °C until use. For immunostaining, slides were dried and fixed in acetone for 5 mins, followed by three 5 min washes in PBS. After blocking with 1% BSA/0.01%NaN_3_ in PBS for 30 mins at room temperature, sections were incubated for 1 hour at room temperature with appropriate antibodies or secondary fluorochrome-conjugated reagent diluted in blocking buffer. Each incubation step was followed by three 5 min washes in PBS. Images were captured on an Axio Imager.A2 microscope (Zeiss).

### Quantitative RT-PCR (qPCR)

Total RNA was isolated with RNeasy Mini Kit (Qiagen) and treated with DNase I (Qiagen). cDNA was prepared with PrimeScript RT reagent Kit (Takara). PCR primers used for real-time quantitative gene amplification are listed in Supplementary Table S4. PCR reactions were run in triplicates using SYBR Premix Ex Taq II (Takara) on a LightCycler480 (Roche). Relative expression levels were normalized to *β-actin* and expressed as mean ±SE.

### Statistical analysis

All statistical analyses were performed with Prism (GraphPad Software 6c) using an unpaired two-tailed *t* test or one-way ANOVA with Tukey’s *post hoc* test. A *P* value of less than 0.05 was considered significant.

## Additional Information

**How to cite this article**: Tan, J. K. H. and Watanabe, T. Stromal Cell Subsets Directing Neonatal Spleen Regeneration. *Sci. Rep.* **7**, 40401; doi: 10.1038/srep40401 (2017).

**Publisher's note:** Springer Nature remains neutral with regard to jurisdictional claims in published maps and institutional affiliations.

## Supplementary Material

Supplementary Information

## Figures and Tables

**Table 1:** Semi-quantitative assessment of 4 week spleen tissue regeneration from single-marker depleted or enriched 3-day old spleen cell aggregates.

Cell marker	Depleted aggregates			Enriched aggregates		
	% +ve cells*	Cells/agg (x10^6^)	4w tissue formation#	% +ve cells	Cells/agg (x10^6^)	4w tissue formation
CD45‡	0.8 (46)	1	+++	93	0.75	−
gp38†	0.03 (0.5)	1	+++	4	1	+++
CD31†	0.04 (1)	1	−	4	1	+++
CD105†	2 (8.4)	1	++	29	1	+++
CD201†	0.9 (3)	1	+	16	1	+++
MAdCAM-1‡	0.3 (1)	1	+	5	0.25	+++
PDGFRβ†	0.1 (1.4)	1	++	11	0.85	+++

^*^Percent positive (+ve) cells before magnetic separation is indicated in brackets.

^#^Tissue formation: Absence (−) or cumulative presence of red pulp (+), multiple T/B cell segregated white pulp follicles (+), marginal zone (+).

^†^Data are representative of 2 independent experiments.

^‡^Data are representative of 3 or more independent experiments.
