# Supplementary material for: Stromal Cell Subsets Directing Neonatal Spleen Regeneration
Source: Sci Rep. 2017 Jan 9;7:40401. doi: 10.1038/srep40401 (PMC5220291; doi:10.1038/srep40401)
Supplement: Supplementary Information [file srep40401-s1.pdf]

Title: Stromal Cell Subsets Directing Neonatal Spleen Regeneration

Authors: Jonathan K.H. Tan and Takeshi Watanabe

SUPPLEMENTAL INFORMATION

Supplemental Tables 1-4

Supplemental Figure 1

Table S1. Marker expression profile of neonatal spleen stromal cell subsets

n.d., not determined

	FRC	MRC	RP stroma	RP sinusoids	CA
CD45	-	-	-	-	-
gp38	+	-	-	-	-
MAdCAM-1	-	+	-	n.d.	-
CD31	-	+	n.d.	-	+
VE-Cadherin	-	+	n.d.	-	+
VCAM-1	lo	hi	-	n.d.	n.d.
ICAM-1	+	hi	n.d.	n.d.	n.d.
MECA-32	-	-	n.d.	n.d.	+
PDGFRβ	-	-	n.d.	n.d.	n.d.
F4/80	lo	lo	+	-	n.d.
CD105	-/+	+	-	+	+
CD201	+	+	n.d.	-	+
CD11b	n.d.	n.d.	-	n.d.	n.d.
FSC	hi	hi	lo	lo	hi

Table S2. Primary antibodies used for cell surface marker binding

Antibody	Alternative Name	Clone	Conjugate(s)	Source
Rat anti-CD5		53-7.3	Biotin	BD Pharmingen
Rat anti-CD11b		M1/70	Alexa Fluor 647	BD Pharmingen
			Biotin	eBioscience
			PECy7	
Rat anti-CD16/32	Fc Block	2.4G2	Unconjugated	BD Pharmingen
		93		BioLegend
Rat anti-CD21/35	CR2/CR1	7G6	PE	BD Pharmingen
Rat anti-CD31	PECAM-1	390	Biotin	eBioscience
			PE	
			Unconjugated	BioLegend
Rat anti-CD45		30-F11	APC eFluor 780	eBioscience
			Biotin	
Rat anti-CD54	ICAM-1	YN1/1.7.4	PE	eBioscience
Rat anti-CD90.2		30-H12	Alexa Fluor 488	BioLegend
Rat anti-CD105		MJ7/18	Biotin	eBioscience
			PE	
Rat anti-CD106	VCAM-1	429	PE	BioLegend
Rat anti-CD140b	PDGFRβ	APB5	Biotin	eBioscience
			PE	
			APC	
Rat anti-CD144	VE-Cadherin	11D4.1	Unconjugated	BD Pharmingen
Rat anti-CD201	EPCR	eBio1560	Biotin	eBioscience
			PE	
Rat anti-CD254	TRANCE	IK22/5	PE	eBioscience
Rat anti-B220		RA3-6B2	Alexa Fluor 488	eBioscience
			Alexa Fluor 647	BioLegend
			PE	BD Pharmingen
Rat anti-ER-TR7		ER-TR7	Unconjugated	Santa Cruz
Rat anti-F4/80		BM8	Biotin	eBioscience
			PE	
Rat anti-FDC-M1		FDC-M1	Unconjugated	BD Pharmingen
Hamster anti-gp38	Podoplanin	eBio8.1.1	Biotin	eBioscience
			PE	
			Unconjugated	AngioBio Co.
Rat anti-Ly6G		1A8	Biotin	BioLegend
Rat anti-MAdCAM-1		MECA-367	Alexa Fluor 488	BioLegend
			Biotin	eBioscience
			Unconjugated	BD Pharmingen
Rat anti-MECA-32		MECA-32	Alexa Fluor 488	BioLegend
			PE	Santa Cruz
Rat $IgG2_a$ Isotype control		eBR2a	APC	eBioscience
			Biotin	
		R35-95	PE	BD Pharmingen
			Unconjugated	
Rat $IgG2_b$ Isotype control		eB149-10H5	Biotin	eBioscience
		A95-1	FITC	BD Pharmingen
			PE	
		eB149-10H5	PECy7	eBioscience

Table S3. Secondary reagents used for flow cytometry and immunofluorescence staining

Antibody	Conjugate(s)	Source
Donkey anti-rat	Alexa Fluor 488	Molecular Probes
	Alexa Fluor 594	
Goat anti-hamster	Alexa Fluor 594	Molecular Probes
Goat anti-rat	Alexa Fluor 647	Molecular Probes
Streptavidin	APC	BD Pharmingen
	DyLight 488	BioLegend
	eFluor 450	eBioscience
	PE	BD Pharmingen
	PECy7	BioLegend

Table S4. Quantitative real time PCR primer sequences

Genes	Forward Sequence	Reverse Sequence
β-actin	catccgtaaagacctctatgccaac	atggagccaccgatccaca
Rank	gctggctaccactggaactc	gtgcagttggtccaaggttt
RankL	attcaggtgtccaacccttcc	tgctaatgttccacgaaatg
Sdf1	gctctgcatcagtgacggta	gggcagcctttctcttcttc
CXCL13	tcgtgccaaatggttacaaa	acaaggatgtgggttgggta
TNFα	cgatgggttgtaccttgtc	cggactccgcaaagtctaag
TNFR1	accaagtgccacaaaggaac	cacgcactggaagtgtgtc
LTa	tccactccctcagaagcact	agagaagccatgtcggagaa
LTBR	gccgaggtcacagatgaaat	caggacactggtgaagagca
PDGFRb	ccggaacaaacacaccttct	tagctgggggactcaatgtc
Ng2	gatccacctcgcatcatctt	gttcccgacaggaaaactca
RelB	ccgtacctggtcatcacagag	cagtctcgaagctcgatggc
ICAM1	actgcttggggaactggac	aggcatggcacacgtatgta
Nkx2.5	aaagaccctcgggcggata	ccatccgtctcggctttgt
Pecam	caacagagccagcagtatgaggac	ctgcaactattaaggtggcgatga
Madcam	cctctgctgagccctacatc	cttgtggtaggttgccaggt
LYVE1	cagcacactagcctggtgtta	cgcccatgattctgcatgtaga
HOX11	aagaagaagccgcgcacatc	ggagtcgtcagaccacggct

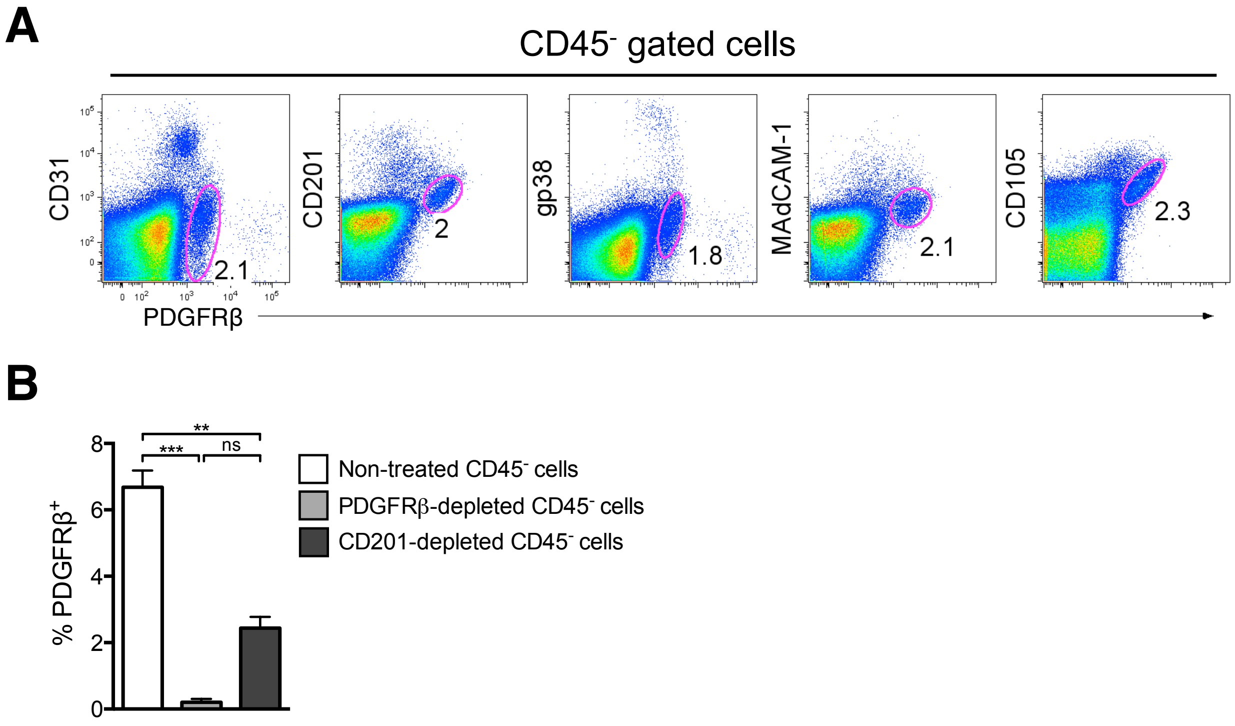

Figure S1. Cell marker analysis of spleen PDGFRβ^{+} cells. (**A**) Neonatal spleen stromal tissue was digested into a single-cell suspension and CD45^{-} gated cells assessed by 2-marker flow cytometry against PDGFRβ expression. Data are representative of three independent experiments. (**B**) Percent PDGFRβ^{+} cells amongst CD45^{-} gated neonatal stromal cell preparations magnetically depleted with PDGFRβ or CD201 antibodies. Error bars indicate SEM. *** $P<0.0001$, ** $P<0.001$; one-way ANOVA with Tukey's *post hoc* test.
